# Additional Observations on Animal Electricity

**Published:** 1792

**Authors:** Eusebius Valli


XX. *Additional Obſervations on Animal Electricity. Communicated in a Letter to* Samuel Foart Simmons, *M. D. F. R. S. by* Euſebius Valli, *M. D.*

SINCE the publication of my two letters in the *Journal de Phyſique*, I have made a conſiderable number of new experiments on animal electricity, many of which you, my dear Sir, and ſeveral of your friends, have had the goodneſs to ſee me repeat, on different days, at your houſe and other places. Theſe additional facts are deſcribed in a ſeries of letters on the ſame ſubject, which before I left Paris I put into the hands of our worthy friend M. De la Metherie, in order to their being inſerted in his valuable Journal. But I ſhall here give you the principal reſults of theſe, and of ſome other ſtill later later inquiries, to be added, if you think proper, to the account of my firſt experiments, which I find you mean to do me the honour of inſerting in the volume of Medical Facts and Obſervations about to be publiſhed.

I now, as you have repeatedly witneſſed, obtain muſcular movements by coating the muſcles inſtead of the nerves.

Movements may likewiſe be excited by eſtabliſhing a communication between muſcle and muſcle, or between nerve and nerve.

By uniting in one coating the nerves of ſeveral frogs, and exciting their electricity, ſome rat's hairs, placed for this purpoſe in the apparatus, were obſerved to recede from and approach each other.

Even in the living frog I am able ſometimes to excite electricity, in a very ſenſible manner, by employing two coatings, one under the belly, and the other on the back.

The different metals, whether employed in the way of coating, or as conductors, afford remarkable differences in their effects. With gold and ſilver, for inſtance, the experiment will, in general, not ſucceed, if one of them be uſed as a coating, and the other as a conductor, though either of them will ſerve as a good conductor when employed with another metal. I have, however, sometimes excited movements in a pullet's wing with silver and gold.

What has been observed concerning the different metals will be found to hold good with respect to different fluids also; some of which, as, for instance, water, will be found to be good conductors; while others will conduct less perfectly, or not at all. It is an observation of Professor Galvani, that oil, which, as we know, is not a conductor of common electricity, is likewise not a conductor of animal electricity.

The experiment in water may be made in the following manner: — The legs of a frog being prepared in the usual way, and the crural nerves coated, two glasses filled with water are to be placed close to each other. The legs of the animal are to be put into one of these glasses, and the nerves with their coating must hang over so as to be immersed in the water in the other glass. You must now, with a piece of silver, or some other metallic conductor, touch the coating of the nerves, while with your other hand you touch the water in the other glass, in which are the legs of the frog; thus forming a communication between the two surfaces of the muscles, cles, and violent movements will inftantly take place. If, inftead of touching the nerves with the conductor, you touch them only with your bare hand, and put the conductor into the water in the other glafs in which are the legs of the frog, no motion will be excited; but if the conductor be made to communicate with the water in both glaffes, it is not neceffary for the fuccefs of the experiment to bring it into contact either with the nerves or with the mufcles. When the electricity of the animal is weak, a conductor made of a fingle metal is not always fufficient to excite it; and it then becomes neceffary to have recourfe to a conductor made of two metals, as, for inftance, of iron and filver.

The membranes of the nerves are bad conductors.

The different intenfity of electricity in different animals will certainly occafion a great difference in its effects.

I have fometimes obferved that frogs have fpontaneous motions ftronger than thofe excited by the metallic conductor; at other times the conductor has failed to produce any movement; but to inveftigate all the various phenomena of animal electricity would require more experiments than I have at prefent leifure to undertake.

This principle has probably a great ſhare in the production of animal heat.

Frogs killed by being plunged into hot water, and immediately taken out of it and prepared, have given ſigns of electricity; but if the limbs of the animal, after being prepared, be placed in very hot water, the experiment does not ſucceed.

The vitriolic and nitrous acids applied to the nerves, or rather to the muſcles, did not prevent the movements.

Opium applied to one of the crural nerves deprived the animal of the power of moving the correſponding leg; but did not prevent movement in it when the conductor was applied to it.

Inflammable, nitrous, and phlogiſticated airs act in different manners both on the living frog and on muſcles that are expoſed to them.

The wings of a pullet, prepared for the experiment, after having given ſeveral ſhocks, remained immoveable, notwithſtanding I continued to apply the conductor; but upon my irritating the nerve ſpontaneous motions immediately took place.

Fowls have little ſenſibility; yet their muſcular power ſeems to be conſiderable. Perhaps the irritability of animals is in an inverſe ratio to that of their ſenſibility.

Fowls killed by drowning have, in general, afforded me ſigns of electricity; and ſome of them, after having been apparently deprived of life in this manner, have been reſtored by exciting their electricity.

Animals that die of hunger have no ſenſible electricity.

Fowls that have died after inflammation or gangrene of the inteſtines have afforded no ſigns of electricity.

The equilibrium of animal electricity is not always broken, notwithſtanding circumſtances which ſeem favourable to ſuch an effect. My experiments on this head are deciſive. I will here mention one of them. I prepared the limb of a frog, and after coating the nerve with lead, placed it on a half-crown piece. At firſt I obſerved ſome movements, but afterwards the limb remained immoveable. By ſhaking the table on which the apparatus was placed, the ſhocks were renewed; but after this, in order to obtain any movement, it became neceſſary to ſhake the coating itſelf. This experiment ſucceeds likewiſe in water.

I have

I have reaſon to think that the nerves contain another fluid which is the vehicle of electricity. My conjecture on this head is founded on different experiments. For inſtance, after having coated a nerve, and apparently exhauſted the electricity of the correſponding limb, if the coating be removed a little higher up to a freſh part of the ſame nerve, movements will again be excited. This ſeems to ſhow that the vehicle only of the electricity in the part, and not the electricity itſelf, is exhauſted.

October 11, 1792.

